# Construction and evaluation of nomogram model for individualized prediction of risk of major adverse cardiovascular events during hospitalization after percutaneous coronary intervention in patients with acute ST-segment elevation myocardial infarction

**DOI:** 10.3389/fcvm.2022.1050785

**Published:** 2022-12-21

**Authors:** Caoyang Fang, Zhenfei Chen, Jinig Zhang, Xiaoqin Jin, Mengsi Yang

**Affiliations:** ^1^Department of Cardiology, The Second People’s Hospital of Hefei, Hefei Hospital Affiliated to Anhui Medical University, Hefei, Anhui, China; ^2^Department of Cardiology, Hefei Second People’s Hospital Affiliated to Bengbu Medical College, Hefei, Anhui, China

**Keywords:** acute ST-segment elevation myocardial infarction, STEMI, percutaneous coronary intervention, PCI, MACE, nomogram model

## Abstract

**Background:**

Emergency percutaneous coronary intervention (PCI) in patients with acute ST-segment elevation myocardial infarction (STEMI) helps to reduce the occurrence of major adverse cardiovascular events (MACEs) such as death, cardiogenic shock, and malignant arrhythmia, but in-hospital MACEs may still occur after emergency PCI, and their mortality is significantly increased once they occur. The aim of this study was to investigate the risk factors associated with MACE during hospitalization after PCI in STEMI patients, construct a nomogram prediction model and evaluate its effectiveness.

**Methods:**

A retrospective analysis of 466 STEMI patients admitted to our hospital from January 2018 to June 2022. According to the occurrence of MACE during hospitalization, they were divided into MACE group (*n* = 127) and non-MACE group (*n* = 339), and the clinical data of the two groups were compared; least absolute shrinkage and selection operator (LASSO) regression was used to screen out the predictors with non-zero coefficients, and multivariate Logistic regression was used to analyze STEMI Independent risk factors for in-hospital MACE in patients after emergency PCI; a nomogram model for predicting the risk of in-hospital MACE in STEMI patients after PCI was constructed based on predictive factors, and the C-index was used to evaluate the predictive performance of the prediction model; the Bootstrap method was used to repeat sampling 1,000 Internal validation was carried out for the second time, the Hosmer-Lemeshow test was used to evaluate the model fit, and the calibration curve was drawn to evaluate the calibration degree of the model. Receiver operating characteristic (ROC) curves were drawn to evaluate the efficacy of the nomogram model and thrombolysis in myocardial infarction (TIMI) score in predicting in-hospital MACE in STEMI patients after acute PCI.

**Results:**

The results of LASSO regression showed that systolic blood pressure, diastolic blood pressure, Killip grade II-IV, urea nitrogen and left ventricular ejection fraction (LVEF), IABP, NT-ProBNP were important predictors with non-zero coefficients, and multivariate logistic regression analysis was performed to analyze that Killip grade II-IV, urea nitrogen, LVEF, and NT-ProBNP were independent factors for in-hospital MACE after PCI in STEMI patients; a nomogram model for predicting the risk of in-hospital MACE after PCI in STEMI patients was constructed with the above independent predictors, with a C-index of 0.826 (95% CI: 0.785–0.868) having a good predictive power; the results of H-L goodness of fit test showed χ^2^ = 1.3328, *P* = 0.25, the model calibration curve was close to the ideal model, and the internal validation C-index was 0.818; clinical decision analysis also showed that the nomogram model had a good clinical efficacy, especially when the threshold probability was 0.1–0.99, the nomogram model could bring clinical net benefits to patients. The nomogram model predicted a greater AUC (0.826) than the TIMI score (0.696) for in-hospital MACE after PCI in STEMI patients.

**Conclusion:**

Urea nitrogen, Killip class II-IV, LVEF, and NT-ProBNP are independent factors for in-hospital MACE after PCI in STEMI patients, and nomogram models constructed based on the above factors have high predictive efficacy and feasibility.

## 1 Introduction

In recent years, the incidence of acute ST-segment elevation myocardial infarction (STEMI) has been increasing, and it has become one of the most common and fatal cardiac emergencies in clinical practice (1). Percutaneous coronary intervention (PCI) is currently one of the most effective treatments for STEMI (2). Although PCI can timely open the infarcted vessel and achieve reperfusion, reperfusion itself aggravates myocardial injury and increases the incidence of major adverse cardiovascular events (MACEs), so it is particularly important to identify high-risk patients with STEMI who have a poor prognosis early in admission (3). Timely risk assessment of STEMI patients has a positive effect on improving patient outcomes. Establishing a convenient and effective prediction model is helpful to assess the risk of in-hospital MACE after emergency PCI in STEMI patients and has a positive effect on the early identification of patients at high risk of in-hospital MACE and timely intervention.

The nomogram is based on the analysis results of COX proportional hazards or logistic regression model, which is graphical and visualized for the prediction of individual disease risk and is more intuitive and easy to be popularized and applied in clinical practice. Compared with traditional risk scoring systems, nomogram models integrate more risk factors and obtain numerical probabilities of target events, more accurately quantify risk, and are more flexible to apply. Its application has been reported in predicting the risk of postoperative heart failure in patients with acute myocardial infarction (AMI) (4), the risk of in-hospital major cardiovascular events in patients with AMI after PCI (5), and the prognosis of patients with the acute coronary syndrome (6). In this study, we retrospectively analyzed the clinical characteristics of 466 STEMI patients before emergency PCI, and provided a reference for clinical assessment of the patient’s condition and guiding treatment by constructing a nomogram model to predict the risk of in-hospital MACE in STEMI patients after emergency PCI.

## 2 Materials and methods

### 2.1 Study population

A retrospective analysis of 466 STEMI patients who underwent emergency PCI at the Second People’s Hospital of Hefei from January 2018 to June 2022 was performed as the study subjects, all of whom were stented patients. Inclusion criteria (1) aged 18 years or older; (2) no previous history of atrial fibrillation; (3) admitted for emergency PCI within 24 h after onset; (4) demographic characteristics and complete clinical data. Exclusion criteria: (1) combined with malignant tumor; (2) accompanied by non-obstructive coronary heart disease, primary cardiomyopathy; (3) clinical evidence of infection; (4) accompanied by immune system disease; (5) combined with severe liver and kidney dysfunction. Patients were divided into the MACE group (*n* = 127) and the non-MACE group (*n* = 339) according to whether MACE occurred in the hospital after PCI. MACE defined the primary endpoint as cardiac death. Secondary endpoints were myocardial reinfarction, malignant arrhythmia, and acute heart failure. Myocardial reinfarction was defined as stent thrombosis in this study. Criteria for stent thrombosis diagnosis were according to those proposed by the Academic Research Consortium (ARC) (7). Diagnosis of acute heart failure: clinical manifestations such as shortness of breath, orthopnea, pulmonary rales, pink foamy sputum; NT-proBNP: >450 ng/L in patients under 50 years old, >900 ng/L in patients over 50 years old, >1,800 ng/L in patients over 75 years old, and >1,200 ng/L in patients with renal insufficiency (glomerular filtration rate <60 ml/min). Malignant arrhythmias include severe sinus bradycardia (≤40 beats/min), high-grade or third-degree atrioventricular block, ventricular tachycardia, ventricular fibrillation, etc., and classify cardiac arrest as a special type of malignant arrhythmia. This study has been approved by the Ethics Committee of the Second People’s Hospital of Hefei (Approval No.: 2020-ke-058). All methods were performed following the Declaration of Helsinki. PCI: refers to the treatment of transcatheter techniques to dredge the stenotic or even occluded coronary lumen, thereby improving the blood perfusion of the myocardium, including percutaneous coronary balloon angioplasty (PTCA), coronary stent implantation, coronary rotational atherectomy, intracoronary thrombus aspiration, and cutting balloon angioplasty.

### 2.2 Study method

#### 2.2.1 Data collection

Demographic characteristics and clinical data of AMI patients at admission were collected through the hospital’s electronic case system, including age, gender, smoking history, heart rate at admission, systolic blood pressure, diastolic blood pressure, comorbidities (including hypertension and diabetes), Killip class II-IV, Gensini score, LVEF of echocardiography results, laboratory parameters (including neutrophils, lymphocytes, hemoglobin, platelets, total bilirubin, direct bilirubin, indirect bilirubin, albumin, triglycerides, total cholesterol, LDL-C, HDL-C, Apolipoprotein B, Apolipoprotein A1, urea, creatinine, uric acid, cystatin C, homocysteine, and fasting blood glucose, NT-ProBNP), Intervention-related data (Gensini score, D-to-B time, infarct location, number of diseased vessels, number of implanted stents, tirofiban, thrombus aspiration, IABP), and MACE data during hospitalization.

#### 2.2.2 Nomogram establishment and verification

Least absolute shrinkage and selection operator (LASSO) regression was used to reduce the dimension of 31 clinical data in this study, predictors of non-zero coefficients were selected, and multivariate logistic regression was used to analyze independent predictors affecting in-hospital MACE after PCI in STEMI patients. Predictors were used to construct a nomogram model to predict the risk of in-hospital MACE after PCI in STEMI patients, and C-index was used to assess the predictive efficacy of the nomogram model for in-hospital MACE after PCI in STEMI patients. Bootstrap multiple sampling 1,000 times was used for model internal validation, model fit was evaluated by the Hosmer-Lemeshow test, and calibration curves were plotted to evaluate the calibration of the model. Decision curves were drawn to analyze the net benefit rate of this nomogram model in predicting in-hospital MACE after PCI in STEMI patients. Receiver operating characteristic (ROC) curves were plotted to assess the efficacy of nomogram models and thrombolysis in myocardial infarction (TIMI) scores in predicting in-hospital MACE after acute PCI in STEMI patients.

#### 2.2.3 Statistical methods

Statistics and graphs were performed using SPSS 26.0, R4.2.1, and GraphPad Prism9.0. Kolmogorov-Smirnov normality test was performed on the measurement data, which conformed to the normal distribution and was expressed as mean ± standard deviation, and an independent sample *t*-test was used for comparison between the two groups; the measurement data without normal distribution were expressed as median M (P25, P75), and Mann-Whitney U test was used for comparison between the two groups; the adoption rate of enumeration data was expressed, and chi-square test was used for comparison between the two groups; LASSO regression was used to select the predictors of non-zero coefficients, and multivariate logistic regression was used to analyze the independent risk factors affecting MACE during hospitalization after PCI in STEMI patients; C-index, area under ROC curve, calibration curve, and clinical decision curve were calculated. All statistics were performed using two-sided tests, and *P* < 0.05 was considered statistically significant.

## 3 Results

### 3.1 Comparison of clinical data between MACE group and non-MACE patients

In this study, there were significant age differences, the proportion of women, history of hypertension, systolic and diastolic blood pressure at admission, neutrophils, hemoglobin, total bilirubin, indirect bilirubin, albumin, urea, creatinine, uric acid, cystatin C, fasting blood glucose, NT-ProBNP, LVEF, Killip class II-IV, Gensini score, number of diseased vessels, thrombus aspiration, and IABP between the MACE group and the non-MACE group (*P* < 0.05). There were no significant differences in diabetes history, smoking history, heart rate, lymphocytes, platelets, direct bilirubin, triglycerides, total cholesterol, LDL-C, HDL-C, Apo-B, Apo-A1, homocysteine, D-to-B time, infarct location, number of implanted stents, and tirofiban between the two groups (*P* > 0.05), as shown in Table 1.

**TABLE 1:** Comparison of general clinical data between major adverse cardiovascular events (MACE) group and non-MACE group.

Variables	MACE group	Non-MACE group	*t*/χ^2^/*z* value	*P*-value
**General clinical data**				
Age (years)^b^	66 (55,78)	59 (51,71)	4.091	<0.001*
Gender (Female, *n* %)	36 (28.35)	64 (18.88)	4.913	0.027*
Diabetes (*n* %)	37 (29.13)	87 (25.66)	0.57	0.45
Hypertension (*n* %)	82 (64.57)	176 (51.92)	5.982	0.014*
Smoking (*n* %)	67 (52.76)	201 (59.29)	1.615	0.204
Heart rate (beats/min)^b^	80 (66.5, 92.25)	76 (67, 86)	1.598	0.11
SBP (mmHg)^a^	112.61 ± 26.3	126.74 ± 22.31	5.79	<0.001*
DBP (mmHg)^a^	68 ± 15.54	77.69 ± 15.43	6.025	<0.001*
Killip grade II-IV (*n* %)	67 (52.8)	60 (17.7)	52.27	<0.001*
**Laboratory data**				
Neutrophils (× 10^9^/L)^b^	8.9 (6.55, 11.77)	7.16 (5.25, 9.68)	4.902	<0.001*
Lymphocytes (× 10^9^/L)^b^	1.3 (0.89, 2.2)	1.49 (1.08, 2.15)	0.974	0.33
Hemoglobin (g/L)^a^	132.26 ± 19.61	138.31 ± 18.4	3.101	0.002*
Platelets (× 10^9^/L)^b^	198.2 (160.75, 241.25)	196.5 (154, 238)	0.892	0.372
Total bilirubin (umol/L)^b^	16.6 (11.88, 21.2)	18 (13.4, 24.68)	2.215	0.027*
Direct bilirubin (umol/L)^b^	4.85 (3.6, 6)	5 (3.7, 6.7)	1.272	0.203
Indirect bilirubin (umol/L)^b^	11.85 (8.28, 15.6)	13.4 (9.6, 17.5)	2.799	0.005*
Albumin (g/L)^a^	37.93 ± 4.11	39.59 ± 3.71	4.164	<0.001*
Triglycerides (mmol/L)^b^	1.42 (0.96, 1.99)	1.51 (1.06, 2.23)	1.628	0.103
Total cholesterol (mmol/L)^b^	4.28 (3.69, 4.98)	4.41 (3.84, 5.11)	1.572	0.116
LDL-C (mmol/L)^b^	2.71 (2.15, 3.3)	2.79 (2.29, 3.42)	1.261	0.207
HDL-C (mmol/L)^b^	1.09 (0.92, 1.29)	1.07 (0.91, 1.24)	0.647	0.518
Apolipoprotein B (g/L)^b^	0.86 (0.71, 0.98)	0.87 (0.74, 1.01)	1.13	0.258
Apolipoprotein A1 (g/L)^b^	1.02 (0.91, 1.18)	1.06 (0.94, 1.18)	1.523	0.128
Urea (mmol/L)^b^	6.4 (5.23, 9.27)	5.15 (4.19, 6.38)	6.653	<0.001*
Creatinine (umol/L)^b^	76 (61.95, 103.88)	69 (58, 78.98)	4.52	<0.001*
Uric acid (umol/L)^b^	373.6 (303.5, 437)	346.8 (281.03, 415.3)	2.535	0.011*
Cystatin C (mg/L)^b^	1.11 (0.91, 1.39)	1.01 (0.88, 1.15)	3.24	0.001*
Homocysteine (umol/L)^b^	14.75 (10.8, 18.17)	13.8 (10.72, 17.4)	0.485	0.628
Fasting blood glucose (mmol/L)^b^	7.37 (6.05, 9.56)	6.06 (5.31,7.78)	5.169	<0.001*
LVEF^b^	56 (48, 61)	60 (56, 64)	5.697	<0.001*
NT-ProBNP (ng/L)^b^	774.42 (466.11, 1071.09)	491.95 (328.67, 731.58)	5.602	<0.001*
**Interventional data**				
Gensini score^b^	80 (42.75, 105)	60 (41, 84)	3.047	0.002*
D-to-B time	61.41 ± 7.88	60.94 ± 7.22	0.608	0.543
Infarct location (*n*, %)	–	–	1.934	0.164
Anterior MI	59 (46.45)	182 (53.69)	–	–
Others	68 (53.55)	157 (46.31)	–	–
Number of diseased vessels (*n*, %)	–	–	3.996	0.046*
1	35 (27.56)	127 (37.46)	–	–
≥2	92 (72.44)	212 (62.54)	–	–
Number of stents implanted^b^	1 (1, 2)	1 (1, 2)	1.294	0.196
Tirofiban, *n* (%)	56 (44.1)	127 (37.46)	1.704	0.192
Thrombus aspiration, *n* (%)	37 (29.13)	53 (15.63)	10.804	0.001*
IABP, *n* (%)	22 (17.32)	9 (2.65)	32.009	<0.001*
**In-hospital MACE**				
Cardiogenic death (*n*, %)	16 (12.6)	–		
Myocardial reinfarction (*n*, %)	2 (1.6)	–		
Malignant arrhythmia (*n*, %)	71 (55.9)	–		
Acute heart failure (*n*, %)	38 (29.9)	–		

SBP, systolic blood pressure; DBP, diastolic blood pressure; LDL-C, low-density lipoprotein cholesterol C; HDL-C, high-density lipoprotein cholesterol C; LVEF, left ventricular ejection fraction; MI, myocardial infarction.

^a^Normally distributed data are expressed as mean ± standard deviation.

^b^Non-normally distributed data are expressed as median M (P_25_, P_75_) 0.1 mmHg = 0.133 kPa; *P < 0.05. Mean ± standard deviation, M (P_25_, P_75_), number of cases and percentage (*n*, %).

### 3.2 Construction of a risk prediction model for in-hospital MACE after PCI in STEMI patients

#### 3.2.1 Predictor variables were filtered by lasso regression

**FIGURE 1:**
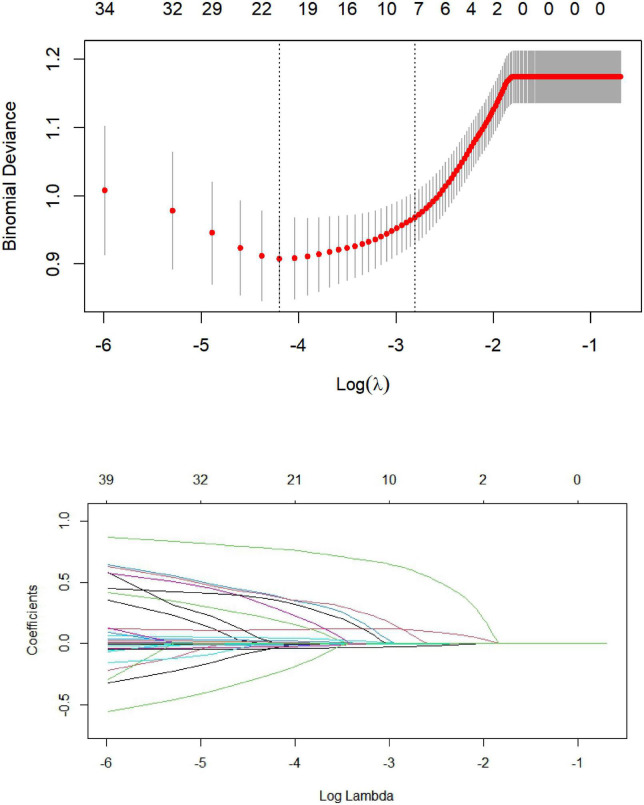
Predictor plots screened by least absolute shrinkage and selection operator (LASSO) regression analysis.

Lasso regression analysis was performed with the presence or absence of MACE (assigned value: NO = 0, YES = 1) during hospitalization after PCI in STEMI patients as the dependent variable and the clinical data and laboratory parameters of the patients as independent variables [categorical variable (assigned value: NO = 0, YES = 1); continuous variable (assigned value: measured value)]. The 39 included variables were dimensionality reduced by Lasso regression, λ values were calculated using 10-fold cross-validation, and finally, λ values within one standard deviation of the least mean square prediction error was selected as optimal values, as shown in Figure. Final Lasso regression analysis screened seven predictors of non-zero coefficients (systolic and diastolic blood pressure at admission, Killip class II-IV, LVEF, urea, NT-ProBNP, IABP) from 39 variables. As shown in Figure 1.

#### 3.2.2 Multivariate logistic regression model construction

**FIGURE 2:**
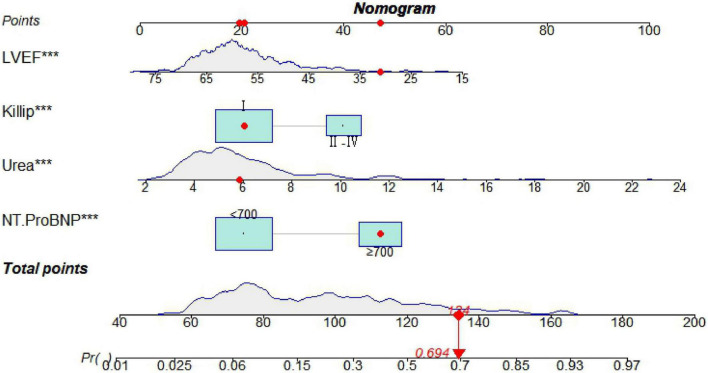
A nomogram predicting in-hospital major adverse cardiovascular events (MACE) after percutaneous coronary intervention (PCI) in patients with ST-segment elevation myocardial infarction (STEMI). ***P < 0.05.

Seven predictive variables, systolic blood pressure (assigned value: measured value), diastolic blood pressure (assigned value: measured value), Killip class II-IV (assigned value: NO = 0, YES = 1), LVEF (assigned value: measured value), urea (assigned value: measured value), and IABP (assigned value: NO = 0, YES = 1), selected by Lasso regression, were used as dependent variables whether MACE occurred during hospitalization after PCI in STEMI patients (assigned value: NO = 0, YES = 1). The optimal Cut-Off value for MACE prediction according to NT-ProBNP was 700 ng/L, with values assigned as 0 for values less than 700 and 1 for values greater than or equal to 700. Multivariate logistic regression models were constructed using these variables as independent variables, and the results showed that Killip class II-IV, urea nitrogen, LVEF, and NT-ProBNP were independent factors for in-hospital MACE after PCI in STEMI patients (*P* < 0.05) as shown in Table 2. A nomogram of the predictive model for the development of in-hospital MACE after PCI in STEMI patients, the Nomogram, was drawn according to the predictive variables and is shown in Figure 2. Each predictor variable corresponds to a specific score on the horizontal axis of the nomogram score, and the scores corresponding to the three predictor variables are summed to obtain a total score. Through the total score corresponding to the risk prediction value of adverse cardiovascular events at the bottom of the nomogram, it can be seen from the figure that the patients with higher total scores are more likely to have in-hospital MACE.

**TABLE 2:** Multivariate logistic regression analysis of influencing factors in major adverse cardiovascular events (MACE) group.

	β	Standard error	*Wald*	OR	95% CI	*P*-value
SBP	0.018	0.009	3.664	0.982	(0.964, 1)	0.056
DBP	0.007	0.015	0.259	0.993	(0.964, 1.021)	0.611
Killip grade II-IV	1.088	0.277	15.411	0.337	(0.922, 0.983)	<0.001*
Urea	0.199	0.055	13.065	0.337	(0.196, 0.58)	<0.001*
LVEF	0.049	0.016	9.03	0.952	(0.922, 0.983)	0.003*
NT-ProBNP	1.485	0.261	32.304	0.226	(0.136, 0.378)	<0.001*
IABP	0.855	0.518	2.726	0.425	(0.154, 1.173)	0.099

SBP, systolic blood pressure; DBP, diastolic blood pressure; LVEF, left ventricular ejection fraction; *P < 0.05.

#### 3.2.3 Nomogram model validation

**FIGURE 3:**
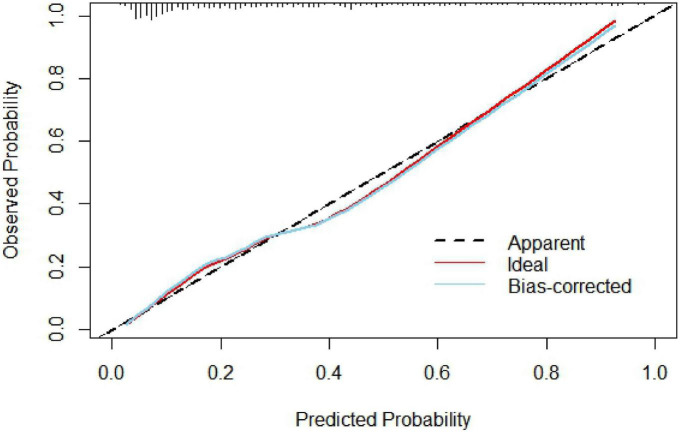
Calibration curve.

**FIGURE 4:**
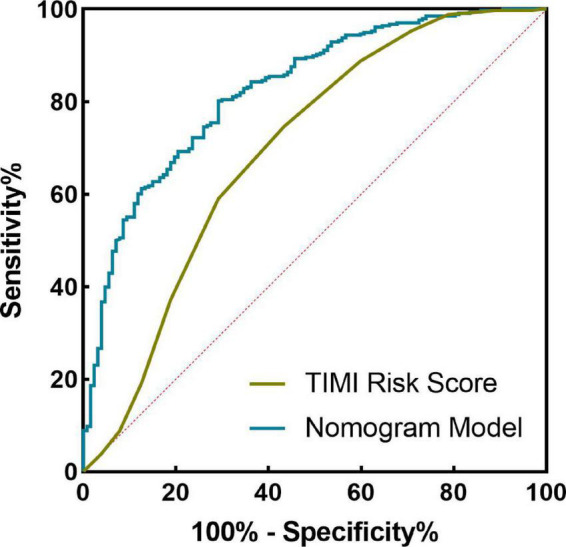
Receiver operating characteristic (ROC) curve of nomogram model and thrombolysis in myocardial infarction (TIMI) score in predicting the efficacy of in-hospital major adverse cardiovascular events (MACE) after percutaneous coronary intervention (PCI) in ST-segment elevation myocardial infarction (STEMI) patients.

The nomogram model concordance index, C-index (equivalent area under the ROC curve AUC), was 0.826 (95% CI: 0.785–0.868); The sensitivity was 0.709, the specificity was 0.802, and the accuracy was 78.7% which had good predictive power. The results of the H-L goodness of fit test of the nomogram model for predicting in-hospital MACE after PCI in STEMI patients showed χ^2^ = 0.44, *P* = 0.51, and the model calibration curve was close to the ideal model, as shown in Figure 3. The internal validated C-index was 0.818 (95% CI: 0.78–0.87), suggesting that the model had good calibration; The Brier score was 0.137, suggesting that the nomogram model predicted in-hospital MACE occurrence in acute ST-segment elevation myocardial infarction with good correlation and strong calibration with internal sampling. ROC curve analysis results showed that the AUC of the nomogram model for predicting in-hospital MACE after PCI in STEMI patients was 0.826 greater than that of the TIMI score 0.696 (Z =3.567, *P* < 0.05) nomogram model had better predictive performance than TIMI score, as shown in Figure 4.

#### 3.2.4 Clinical decision analysis for nomogram models

**FIGURE 5:**
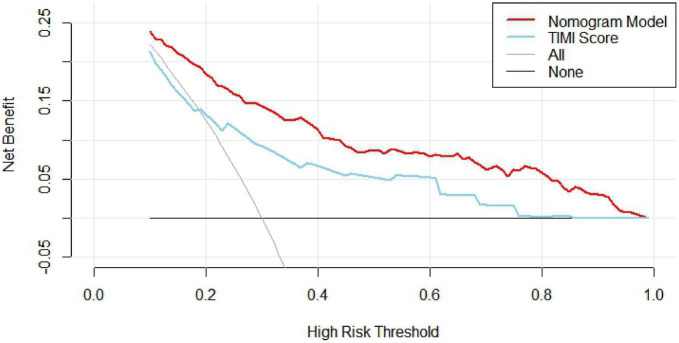
Clinical decision curve analysis for nomogram models.

**FIGURE 6:**
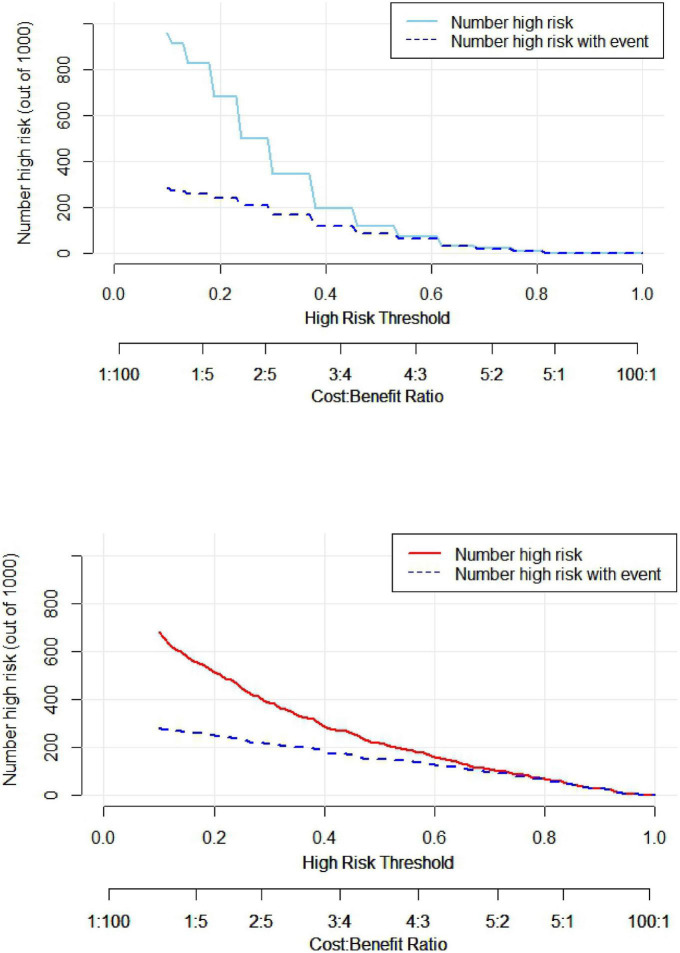
Analysis of clinical impact curve between nomogram model and TIMI score single model.

The clinical decision curve (DCA) was plotted with the probability of the high-risk threshold as the abscissa and the net benefit rate as the ordinate, in which the probability of the high-risk threshold was set at (0, 1), the black solid line represented the net benefit rate of in-hospital MACE in all patients, the gray solid line represented the net benefit rate of in-hospital MACE in all patients, the sky blue curve represented the single model decision curve taking TIMI score as an example, the red curve represented the decision curve of this nomogram model, which was positive or negative relative to all study subjects, and the nomogram predicted the net benefit of the model in the interval of 0.1–0.99 with a threshold probability of 0.1–0.99, suggesting that the nomogram model could bring net clinical benefit to patients when the threshold probability was 0.1–0.99, as shown in Figure 5. The clinical impact curve (CIC) can further reflect the use of the nomogram model to predict the risk stratification of 1,000 people, showing the coordinate axis of “loss: benefit,” assigned with eight scales, green in the figure is a single factor model represented by TIMI score, red is a nomogram model, gray represents the actual occurrence of in-hospital MACE, it is seen that compared with the single model of TIMI score, the difference between the nomogram model curve and the actual occurrence curve is smaller, suggesting that the nomogram model is more suitable for the actual occurrence of in-hospital MACE in clinical practice, as shown in Figure 6.

## 4 Discussion

This study aimed to investigate the occurrence of MACE during hospitalization in patients with STEMI, so the primary study endpoint in the definition of MACE was cardiac death. Secondary endpoints included myocardial reinfarction, first acute or subacute thrombosis, which could be induced at the site of stent implantation due to intimal injury. In addition, when interventional therapy is performed for major vessels, it can compress branch vessels to a certain extent, resulting in vascular occlusion, and also causing some myocardial necrosis with symptoms of acute myocardial infarction. During the onset of acute myocardial infarction, various arrhythmias, especially ventricular arrhythmias, can occur due to myocardial ischemia, and in severe cases, cardiorespiratory arrest in patients. In patients with inferior myocardial infarction, inhibition of the sinoatrial node and atrioventricular node function can lead to heart rate reduction and atrioventricular block. For patients with longer ischemic events, due to more myocardial cell necrosis, patients still cannot save the necrotic myocardium after opening the vessel, resulting in decreased cardiac pump function during hospitalization, resulting in acute heart failure or even cardiogenic shock. Therefore, in this study, these indicators were selected as meeting the endpoints, and predictors were selected to construct a nomogram model to provide a reference for clinical assessment of the patient’s condition and guiding treatment.

Acute myocardial infarction (AMI) is a serious and fatal disease with high mortality and poor prognosis. Although PCI can restore myocardial perfusion associated with an infarcted artery as soon as possible and improve the prognosis of patients, the risk of major adverse cardiovascular events in patients after PCI is still very high, mainly including cardiac death, heart failure, stroke, revascularization, malignant arrhythmia, etc. (8–10). Studies have shown that poor prognosis after PCI in patients with acute myocardial infarction is associated with several indicators, such as LVEF, Killip class, Hb, and red blood cell distribution width (11–13).

Therefore, early assessment of short-term risk in STEMI patients, including assessment of the extent of myocardial injury, presence of clinical features at high risk of developing MACE, risk of reperfusion therapy, and success, is important. To obtain more effective prognostic information, the scoring system for patient risk assessment mainly includes the TIMI risk score (14), GRACE risk score (15), PAMI risk score (16), etc., of which TIMI risk score is widely used in clinical practice (14). The TIMI risk score was based on data from the TIMI-II study, which enrolled STEMI patients who presented within 6 h and underwent thrombolytic therapy, taking into account risk factors and reperfusion time, and therefore has important prognostic value for STEMI patients, especially STEMI patients receiving reperfusion therapy. The GRACE risk score model was derived from the Global Registry of Acute Coronary Events (17), which primarily enrolled patients with non-ST-segment elevation acute coronary syndrome, and the score was used to risk stratify patients based primarily on their basic clinical signs and ancillary tests and did not include the impact of reperfusion on prognosis. Therefore, it is generally believed in clinical work that the TIMI risk score is more accurate for risk stratification of STEMI patients, while the GRACE risk score is more reasonable for risk stratification of non-ST-segment elevation acute coronary syndrome patients. Therefore, early screening of patients at high risk of MACE and individualized management are beneficial to improve patient outcomes.

To better individualize patient outcomes, we used Lasso regression to screen five risk factors most associated with major adverse cardiovascular events during hospitalization: systolic blood pressure (SBP) and diastolic blood pressure (DBP) at admission, Killip class II-IV, urea, left ventricular ejection fraction (LVEF), IABP, and NT-ProBNP as significant predictors with coefficients that were not zero. Previous studies have shown that blood pressure at admission has also been shown to be a risk factor for in-hospital outcomes in STEMI patients. Acute myocardial infarction patients with hypertension have a poor prognosis (18), while other studies have shown that AMI patients with low SBP and low DBP levels at admission are significantly associated with the risk of in-hospital mortality (19, 20). In this study, we found that SBP and DBP at admission were lower and statistically significantly different in patients affecting the MACE group than in the non-MACE group. We believe that lower SBP and/or lower DBP impacts myocardial perfusion and thus adversely impacts prognosis. In this study, multivariate logistic regression analysis was performed with five predictor variables SBP, DBP, Killip class II-IV, LVEF (assigned value: measured value), and urea selected by Lasso regression as independent variables, and the results showed that Killip class II-IV and urea were independent risk factors for in-hospital MACE after PCI in STEMI patients (*P* < 0.05), and LVEF was an independent protective factor for in-hospital MACE after PCI in STEMI patients (*P* < 0.05).

Killip classification is an index to assess the severity of heart failure after acute myocardial infarction (21). Previous studies have reported that myocardial infarction patients with higher Killip grades tend to have more severe coronary artery disease and larger myocardial infarct size, which means more myocardial cell necrosis and necrotic cells are subsequently replaced by fibrotic scars, which are difficult to reverse once formed, in addition to their effects on cardiac contractility, which interfere with normal cardiac electrical activity and thus lead to arrhythmia, which may be a factor in the poor long-term prognosis of patients with higher Killip grades (22, 23). At the same time, DeGeare et al. (24) reported that patients with a higher Killip class were more likely to develop renal failure after PCI, and some patients required long-term dialysis therapy, which may also be partly responsible.

Patients with acute myocardial infarction complicated by heart failure (HF) or left ventricular dysfunction have a poor prognosis and are at high risk of rehospitalization and death (25). Assessment of left ventricular function using echocardiographic measurements of LVEF after acute myocardial infarction is an important predictor of clinical outcome (26) and can well distinguish between low and high risk of cardiac events after acute myocardial infarction. In a study of 417 patients with AMI, LVEF <40% was an independent predictor of the combined end point of death, congestive heart failure, and recurrent AMI 30 years after AMI (27). In another large prospective cohort study (28), 4,122 patients with acute myocardial infarction undergoing PCI were followed up for 4 years and found to have a significantly increased risk of sudden cardiac death and all-cause mortality in patients with LVEF ≤30 and 30< LVEF ≤40% compared with those with LVEF >40%. Another study (29), involving 28,771 patients with HF, left ventricular dysfunction, or both after acute myocardial infarction, showed that the risk of death increased with decreasing LVEF for all types of death.

NT-proBNP is an endogenous hormone produced by ventricular myocytes, and it has been shown that its peripheral content is not significantly changed in the early stages of ventricular dysfunction, but is significantly increased in patients with acute heart failure (30). In clinical practice, LVEF, NYHA functional classification, A-D stage, and other indicators are the main indicators to determine heart failure, but these indicators have a certain subjective color, in reflecting the severity of heart failure, it is bound to be subjectively affected, resulting in a certain degree of inaccuracy. NT-proBNP levels have been reported to more accurately reflect the severity of heart failure and correlate well with NYHA functional class (31). When acute heart failure occurs, NT-ProBNP levels rise dramatically in the patient’s plasma. NT-ProBNP levels have a close correlation with left ventricular systolic dysfunction (30, 32) and can be used as a sensitive indicator to determine the ventricular function and the degree of cardiac insufficiency, as well as to evaluate the clinical treatment effect and prognosis (33, 34). This study also found that serum NT-proBNP was significantly increased in patients who developed acute heart failure, consistent with the trend in the above studies.

Previous studies have shown that renal dysfunction in STEMI patients is one of the most important predictors of in-hospital and long-term mortality (35). And serum creatinine levels are closely related to prognosis after treatment (36). This study showed that creatinine and urea levels were significantly higher in the MACE group than in the non-MACE group (*P* < 0.05), while elevated blood urea nitrogen levels were independent risk factors for in-hospital MACE in STEMI patients. Early restoration of effective myocardial reperfusion in STEMI patients is critical to reducing acute mortality and improving prognosis; however, interventional or medical therapy is often limited by renal function and serum creatinine levels. Therefore, serum creatinine and urea nitrogen levels should be used as important predictors of prognosis when individualizing treatment regimens, and risk stratification should be performed according to renal function status as well as blood urea nitrogen and creatinine levels, which ultimately effectively reduces mortality and improves hospital outcomes.

A nomogram is a visual graph composed of line segments of different lengths that are used to predict the probability of a clinical event, is based on a multivariate regression model, and is drawn after integrating multiple clinical indicators. In this study, we constructed a nomogram model for risk prediction of in-hospital MACE after PCI in STEMI patients based on indicators that were statistically different in multivariate logistic regression analysis. The results showed that the AUC of the nomogram model for predicting in-hospital MACE after PCI in STEMI patients was greater than that of the TIMI score, indicating that the nomogram model constructed in this study had a higher predictive value for in-hospital MACE after PCI in STEMI patients compared with TIMI score. The nomogram model concordance index (C-index) was 0.826 (95% CI: 0.785–0.868), with a sensitivity of 0.709 and a specificity of 0.802, which had good predictive power. The results of the H-L goodness-of-fit test for predicting in-hospital MACE after PCI in STEMI patients showed χ^2^ = 1.3328, *P* = 0.25, and the model calibration curve was close to the ideal model, with an internally validated C-index of 0.818 and good discrimination. Clinical decision curve (DCA) analysis showed that the net benefit of the nomogram prediction model was higher in the interval of 0.1–0.99 for threshold probability, suggesting that when the threshold probability was 0.11–0.99, the nomogram model could bring net clinical benefit to patients; clinical impact curve (CIC) analysis showed that compared with the TIMI score single model, the difference between the nomogram model curve and the actual disease curve was smaller, suggesting that the nomogram model was more suitable for the actual occurrence of in-hospital MACE in clinical practice, and the prediction model judged that STEMI patients at high risk of in-hospital MACE were highly matched with STEMI patients who developed in-hospital MACE, confirming that the prediction model had a high clinical effective rate.

This study still has shortcomings: firstly, this study is a single-center study with a limited sample size, the risk factors included in the study are not comprehensive and bias cannot be avoided; secondly, in terms of model validation, only internal validation has been performed. Third, due to the limitation of our medical institution level, all enrolled patients in this study were patients who underwent coronary artery stenting, so the effect of different PCI procedures on MACE still needs further study. Lack of external validation results from other sites. Therefore, in terms of the clinical application and promotion of this model, large-sample, multicenter clinical data are still needed to provide more external evidence to support further exploring the influencing factors of in-hospital MACE after PCI in STEMI patients and optimize the nomogram model.

## 5 Conclusion

In summary, Killip class II-IV, urea nitrogen, and LVEF NT-ProBNP are independent factors for in-hospital MACE after PCI in STEMI patients, and a nomogram model for in-hospital MACE risk prediction after PCI in STEMI patients constructed based on the above factors has good discrimination, calibration, and clinical effectiveness and can be used as an effective tool for early clinical prediction of in-hospital MACE risk after PCI in STEMI patients.

## Data availability statement

The raw data supporting the conclusions of this article will be made available by the authors, without undue reservation.

## Ethics statement

The studies involving human participants were reviewed and approved by the Second People’s Hospital of Hefei Ethics Committee. Written informed consent for participation was not required for this study in accordance with the national legislation and the institutional requirements.

## Author contributions

CF and ZC wrote the main manuscript text. XJ and MY prepared Tables 1, 2 and Figures 1–6. All authors reviewed the manuscript and approved the submitted version.
